# Genetic variation associated with side effects of hormonal contraception exposure: a narrative review

**DOI:** 10.3389/frph.2025.1720994

**Published:** 2025-11-14

**Authors:** Mariah Nuzzo, Elise N. Erickson, Susan W. Groth, Yang Yu, Theresa Koleck, Huashi Li, Kiana Martinez, Adnin Zaman, Caitlin Dreisbach

**Affiliations:** 1 Goergen Institute for Data Science, University of Rochester, Rochester, NY, United States; 2 Department of Physiology College of Medicine Tucson, University of Arizona, Tucson, AZ, United States; 3 School of Nursing, University of Rochester, Rochester, NY, United States; 4 School of Nursing, University of Pittsburgh, Pittsburgh, PA, United States; 5 Statistics Consulting Lab, BIO5 Institute, University of Arizona, Tucson, AZ, United States; 6 Department of Pharmacy Practice and Science, The University of Arizona R. Ken Coit College of Pharmacy, Tucson, AZ, United States; 7 Division of Endocrinology, Diabetes and Metabolism, Department of Medicine, University of Rochester Medical Center, Rochester, NY, United States

**Keywords:** pharmacogenomics, contraception, symptoms, side effects, single nucleotid polymorphism (SNP)

## Abstract

Hormonal contraceptives (HCs) are commonly prescribed medications that have had immeasurable impacts on quality of life and health of women and families globally. However, usage of exogenous hormones is not without risks, and patients often report a variety of side effects, ranging from burdensome to life-threatening. For some patients, side effects of HCs are severe enough to cause medication discontinuation or switching to alternative forms of contraception. Variability in side effect profiles may indicate heritable risk factors for some side effects. Understanding these patterns or risk profiles may help clinicians anticipate severe adverse events, match patients with suitable medications more rapidly, and improve patient outcomes and adherence. To support further research in this field, this narative review summarizes what is currently known about pharmacogenetic interactions with respect to HCs and specific polymorphisms suspected to contribute to adverse side effects and outcomes.

## Introduction

Hormonal contraceptives (HCs) offer major benefits for many individuals, including control over family planning and pregnancy, regulation of menses, and improvement in numerous health conditions (e.g., endometriosis, dysmenorrhea, abnormal uterine bleeding, acne). HCs, medications intended to decrease the risk of pregnancy following sexual intercourse, contain a progestin with or without an estrogen (1). By introducing exogenous versions of these hormones at levels significantly higher than baseline, HCs can affect multiple body systems and produce numerous side effects in addition to pregnancy prevention (1–3). Patient response to HCs is highly variable, ranging from positive side effects (such as improved health and mood) to severe negative side effects such as depression, hypertension, stroke, and venous thromboembolism (4–6). Due to dissatisfaction with their current HC method, many women discontinue HCs despite wanting to prevent pregnancy (7). The majority of women cite side effects such as breakthrough bleeding, weight gain, or mood changes as the cause for discontinuation (5, 8, 9). Switching or discontinuing these medications can lead to unwanted pregnancies, as many women who discontinue HCs take up less reliable methods, use barrier methods inconsistently, or report no contraceptive methods at all (5, 9).

Emerging evidence suggests that genetic variation may play a role in individual susceptibility to certain adverse effects of HC, although this area remains underexplored (10). Due to the variability of responses to HC exposure and the familial tendencies of some adverse effects, it has been postulated that genetics may influence HC effects (10). No studies to date have systematically evaluated whether genetic factors (e.g., single nucleotide polymorphisms, epigenetic changes) can be used as a predictive tool for hormonal contraceptive side effects, and limited research has been focused on the effects of genotype on HC response. The purpose of this narrative review is to identify side effect profiles associated with HCs and single nucleotide polymorphisms (SNPs) that are known, or suspected, to alter these phenotypic responses. We summarized side effects that have been reported as common reasons for discontinuation of HCs and have been studied in conjunction with genetic risk factors (Table 1). We aim to identify trends in genetic susceptibility to these side effects, which will aid in prediction of adverse reactions, offer a pathway toward more individualized, risk-informed contraceptive counseling, and highlight gaps remaining in research.

**Table 1:** A summary of polymorphisms studied in relation to altered hormonal contraceptive response. Polymorphisms with evidence of positive interaction effects are highlighted in green.

Gene	Polymorphism	Trait	No. patient	No. Control	Population^a^	Contraceptive agent	Main findings	Study
ESR1	rs9340799	Weight Gain	276	N/A	Hispanic, Latina, white	Etonogestrel implant	Homozygous carriers gained on average 14.1 kg more weight during etonogestrel implant use, this difference was statistically significant.	Lazorwitz et al. (10)
CYP2C19	rs7088784	Weight Gain	276	N/A	Hispanic, Latina, white	Etonogestrel implant	Carriers of CYP2C19 rs7088784 variant had less weight gain compared to the wild-type genotype, this was not significant after multiple-testing correction.	Lazorwitz et al. (10)
GCH1	rs8007267, rs3783641, rs10483639	Pain sensitivity in PVD	98	102	Swedish	Mixed, hormonal contraceptives	No association of GCH1 SNPs with PVD diagnosis or pain. Among patients currently under PVD treatment, HC use interacted with GCH1 variants to influence pain perception.	Heddini et al. (19)
ABCB11	rs2287622 (1331T > C)	Intrahepatic and contraceptive-induced cholestasis (ICP & CIC)	42 (ICP), 4 (CIC)	245* (40 pregnant)	Caucasian	Mixed COC (30–35 μg ethinylestradiol)	1331C allele significantly overrepresented in ICP and CIC patients. All CIC patients were homozygous CC, bile acid levels increased with number of C alleles.	Meier et al. (18)
ABCC2	rs17222723 (3563T > A), rs8187710 (4544G > A)	ICP & CIC	33 (ICP), 4 (CIC)	152	Caucasian	Mixed COC (30–35 μg ethinylestradiol)	No statistically significant association between ABCC2 polymorphisms and ICP or CIC.	Meier et al. (18)
CYP17A1	rs743572	Endometriosis risk	119	108	Taiwanese	None	The T -> C polymorphism was less common in the group of women with endometriosis, the wild type was proportionally more common in the group with endometriosis	Hsieh et al. (17)
ESR1	TA repeats 1,174 bp upstream of gene	Endometriosis risk	119	108	Taiwanese	None	14, 18, or 24 TA repeats were associated with endometriosis	Hsieh et al. (17)
CLOCK	rs1801260 (T3111C,)	Startle response (and circadian rhythm)	Study 2: 50	Study 2: 58	N/A	Varied (COC)	CLOCK T3111C, typically linked with circadian rhythms and startle response, appeared to interact with hormone status, causing differential physiological stress responses between free-cycling participants and COC users.	Armbruster et al. (21)
MR haplotype (NR3C2)	rs2070951 (MR-2G/C) and rs5522 (MR-I180 V A/G)	Emotional processing	44 taking OCs	41 NC	Western European	Varied (OC)	OC-mediated effect on facial expression recognition, emotional memory and decision-making was demonstrated. Carriers of MR haplotype 1 or 3 were sensitive to the impact of OCs on the recognition of sad and fearful faces and on emotional memory, whereas MR haplotype 2 carriers were not	Hamstra et al. (2)
MR haplotype 2 (NR3C2 gene)	rs2070951 (MR-2G/C) and rs5522 (MR-I180 V A/G)	Mood and cognition	57 (using OCs)	35 NC	North-western European	Ethinyl estradiol (EE; 0.03)/levo-norgestrel (LNG; 0.15)	OC users were found to have fewer mood swings and lower scores for reproductive depression. MR haplotype had mild effects on the influence of hormonal status on mood, but these were not significant after correction for multiple comparison.	Hamstra et al. (3)
FKBP5	rs1360780	Stress response	74	159	German	Mixed OCs, primarily COCs	OC use is associated with increased circulating cortisol, alterations in circulating lipid levels, and increased FKBP5 expression. FKBP5 expression, methylation, and phospholipid alterations were modified by rs1360780 genotype	Hertel et al. (20)
ESR2	rs1256049 (G1082A)	Hypertension	621	621	Chinese	COC	Heterogenous (GA) individuals were at an increased risk of hypertension. Cumulative duration of COC use was associated with hypertension risk. GA carriers with COC exposure were at a higher risk of hypertension.	Chen et al. (22)
ESR2	rs4986938 (G1730A)	Hypertension	621	621	Chinese	COC	No significant association found	Chen et al. (22)
AGT	rs699 (T235)	Hypertension	149	101	N/A	Oral Contraceptives	Women were split into groups of normotensive, OC-induced hypertensive, and latent essential hypertensive patients. The 235 T allele was more frequent in the OC—induced hypertensive group.	Mulatero et al. (11)
AGT	Promoter region variant	Hypertension	149	101	N/A	Oral Contraceptives	No significant association found	Mulatero et al. (11)
ACE	I/D	Hypertension	149	101	N/A	Oral Contraceptives	No significant association found independently (although non-random distribution of genotypes noted when stratified by AGT genotype)	Mulatero et al. (11)

a Population descriptors based on articles where provided.

## Weight gain

One side effect most associated with HCs, particularly DepoProvera or medroxyprogesterone acetate (12), is weight gain. Providers are recommended to counsel women about the possibility of weight change prior to new HC medication and weight changes are frequently cited as a concern or reason for discontinuation (5, 6, 10, 13–15). Despite anecdotal data, the risk of weight gain remains heavily debated in the literature, with many researchers questioning the link between HCs and weight gain given the lack of randomized controlled trials and the tendency for weight to fluctuate naturally over time (6, 13, 14). Multiple studies with patient-control groups have found no statistically significant differences between weight outcomes, however, these studies also show that outcomes at the individual level vary widely (13, 15). The variation may suggest a multifactorial source of weight change including possible genetic predispositions which make some patients more likely to gain weight once introduced to exogenous hormones (10, 15). In one retrospective analysis, Lazorwitz et al. considered 99 genetic variants among reproductive-aged women with an etonogestrel contraceptive implant placed 12–36 months prior (Table 2) (10). Current weight was compared to pre-insertion weight using data from medical records. One SNP on the estrogen receptor 1 (*ESR1*) gene (rs9340799) was found to have a statistically significant association with weight change, with participants homozygous for the variant (genotype GG) gaining 14.1 kg more weight on average than those with at least 1 wild-type (A) allele (10). Although etonogestrel is not known to bind to estrogen receptors, progesterone receptor activation is suspected of altering *ESR1* transcription, and the authors posit exogenous progesterone sources may affect estrogen receptor protein production or action (10).

**Table 2:** A summary of rationale given in referenced articles as to why selected polymorphisms were studied .

*Gene* (protein)	SNPs	Why these genes were chosen	Author
*CYP2C19*	rs7088784	This was a retrospective study using data from prior research on 120 genetic variants in 14 genes encoding proteins involved in steroid hormone metabolism, regulation, or function. Of the 99 variants observed in this study's population, 7 genetic variants were found to have significant associations with weight gain, these 2 remained when adjusting for BMI on enrollment. Only the *ESR1* gene was significant after Bonferroni correction.	Lazorwitz et al. (10)
*ESR1*	rs9340799		
*GCH1*	rs8007267 rs3783641 rs10483639	Prior studies had identified this set of polymorphisms on the GCH1 gene as pain protective. GCH1 is a rate limiting enzyme in the cofactor of several pain modifiers.	Heddini et al. (19)
*ABCB11* (BSEP)	rs2287622 (1331T > C)	The Bile Salt Export Pump (BSEP) and multidrug resistance protein 2 (MDRP2) proteins were previously proposed as candidates involved with hormonal cholestasis. The SNP in the ABCB11 gene was previously found to be overrepresented in populations with drug induced cholestatic liver injury and ICP. The ABCC2 SNP affects the expression levels of MRP2 which is a bilirubin transporter.	Meier et al. (18)
*ABCC2* (MRP2)	rs17222723 (3563T > A), rs8187710 (4544G > A)		
*ESR1*	TA repeats in promoter	Previous studies showed certain numbers of TA repeats were more prevalent in patients with endometriosis. Estrogen receptor function suspected of contributing to pathology.	Hsieh et al. (17)
*CYP17A1*	rs743572 (A1/A2 promoter region polymorphism)	This gene encodes a protein involved in estrogen biosynthesis. Some prior studies had associated the polymorphism with estrogen-mediated diseases such as breast cancer and polycystic ovarian syndrome.	
*CLOCK*	rs1801260 T3111C	This study was interested in alterations in circadian rhythms and mood. This SNP had previously been found to be linked with dysregulated sleep in some patient populations and potentially to development of numerous psychiatric disorders. The authors note that estrogen affects circadian rhythms and they hypothesized hormone status could explain variance in physiological parameters.	Armbruster et al. (21)
*NR3C2* (MR)	rs2070951 rs5522	These SNPs classify mineralocorticoid receptor (MR) haplotype. The authors note variability in the depressogenic effects or depression-protection for OC users. They cite animal studies showing estrogen and progesterone affect MR binding and that MR is tied to mood and stress response and hypothesize that synthetic hormones effects on mood might be moderated by different MR haplotypes	Hamstra et al. (2)
*NR3C2* (MR)	rs2070951 rs5522	The team again looks at the interaction of sex steroids and the HPA axis by comparing MR haplotype with hormone status	Hamstra et al. (3)
*FKBP5*	rs1360780	This team examines COC effect on the HPA axis. *FKBP5* encodes a protein regulator of the HPA axis. This SNP is associated with alterations in the expression level of FKBP5, the T allele is associated with increased expression and depression risk when combined with childhood trauma. Because OCs are associated with higher levels of circulating cortisol, the authors test if the combination of Ocs and the SNP cause stress-like alterations in gene expression.	Hertel et al. (20)
*ESR2*	rs4986938	ESR2 has a role in vascular function and blood pressure regulation and previous studies had shown polymorphisms linked to cardiovascular disease and hypertension. Per references, previous studies had associated these SNPs with coronary artery disease and cardiovascular disease respectively.	Chen et al. (22)
*ESR2*	rs1256049		
*AGT*	rs699 (T235M)	There is evidence showing the renin-angiotensin system is activated in OC-induced hypertension but it is unclear how estrogen interacts with it and if there are protective genetic factors. The T235M polymorphism was previously associated with preeclampsia and essential hypertension. Polymorphisms in the promoter region of the angiotensinogen gene have been associated with essential hypertension. Alleles in the ACE insertion/deletion polymorphism were associated with cardiovascular disease.	Mulatero et al. (11)
*AGT*	Promoter region		
*ACE*	Insertion/Deletion		

Including genomic considerations into analyzing weight gain in the setting of contraceptive use appeared to explain a substantial amount of variance compared with studies focused on all HC users. A genetic predisposition to weight change could markedly impact a patient's outcome, and their choice to take or continue a medication. While these results are significant, we found this field to be understudied. Additional research would be warranted to confirm these results and examine relevance to alternative sources of exogenous hormones.

## Abdominopelvic symptoms

Abdominal and pelvic pain are common complaints with HC use, particularly with oral contraceptive pills (5, 9). Much of the literature describing abdominal pain with HC use focuses on specific patient groups with, or at risk for, pre-existing conditions such as endometriosis. Consequently, the study of abdominal pain, HCs, and genotype interactions is broad and tends to be condition specific.

Estrogen itself may play a primary role in the effect of HCs on abdominal and GI symptoms as it has been implicated in multiple “estrogen-dependent” diseases. Endometriosis, as an example, is an estrogen-dependent disease with strong familial tendencies which is associated with severe pelvic pain and dysmenorrhea (16, 17). Estrogen-dependent conditions may be particularly affected by combined hormonal contraceptives, as some clinicians have concerns that high doses of estrogen may cause progression in these conditions, making them more severe or difficult to treat (16). Preliminary genomics studies have found associations between endometriosis and polymorphisms in the *ESR1* and *CYP17A1* genes. However, the interaction between these polymorphisms and contraceptives has not yet been examined and may benefit from further research.

A more direct connection can be found between HCs and the development of oral contraceptive-induced cholestasis (CIC), an acquired form of cholestasis characterized by impaired bile flow from the liver to the duodenum. CIC is known to resolve after discontinuation of HCs and has been suspected of genetic origins due to regional clustering and similarity to intrahepatic cholestasis of pregnancy, which has known familial tendencies (18). In a study of CIC and intrahepatic cholestasis of pregnancy, Meier et al. found higher frequency of the *ABCB11* 1331T > C polymorphism (rs2287622) in patients with ICP or CIC than in pregnant controls. Although this study was limited due to the small population of patients (with only four CIC patients included), it may offer preliminary evidence of the role of the *ABCB11* bile salt export pump gene in the development of CIC. Further work in these conditions could support to the theory that certain individuals could be at elevated risk when starting exogenous steroid hormones, although the etiology of CIC is likely multifactorial and joint risk factors would need to be considered to establish a full risk profile.

While estrogen-dependent disease progression may be mediated by HCs due to the introduction of exogenous hormones, there is additional evidence that HC use can alter pain sensation overall or modify pre-existing conditions through interactions with other genotypes. Research on provoked vestibulodynia, a chronic pain condition characterized by pain localized to the area surrounding the vaginal opening, found an interaction between hormone status, pain, and SNPs on the *GCH1* gene. This gene codes for a rate limiting enzyme for B4, a cofactor in pain modulator synthesis. A 15 SNP combination on the *GCH1* gene has been identified as protective and can be identified by genotyping as few as 3 SNPs: rs8007267, rs3783641, and rs10483639 (19). When studied in women currently receiving treatment for vestibulodynia, a treatment-hormonal interaction emerged. Carriers who did not take HCs reported lower coital pain and higher pressure pain thresholds on the arm while patients using HCs noted the reverse, with carriers reporting more pain and lower pain thresholds than non-carriers (19). The reversal may indicate that some aspect of exogenous hormonal medication blocked protective pathways of inherited polymorphisms, making these participants more likely to develop or notice worsening symptoms of pre-existing conditions after HC use was initiated. The authors speculated that this could explain the tendency for some patients to do better when taken off HCs, supporting the concern for frequent discontinuations or medication switching in this group (19).

While numerous diseases may depend on gene-environment interactions resulting in alterations in abdominal, pelvic, or vulvar pain attributed to HC use, there is very limited coverage in the literature, with conclusions muddled by contradictory results. While it is difficult to determine how patients of a given genotype may respond to specific hormonal medications given the paucity of research, there is evidence that multiple genes, perhaps most notably those responsible for mediating effects of sex steroids, may mitigate or worsen disease and symptom progression. Additional research may be able to further compare the risks or benefits of different HCs for these patients or investigate contraceptive users as a whole rather than specific patient groups.

## Mood, energy, and emotional effects

Along with physiological changes, HCs have been studied as having numerous psychiatric side effects, ranging from depressogenic to depression-protective in different patients (2). Depression and mood-related side effects are frequently reported following new HC prescriptions, and are commonly cited causes for discontinuation (2, 4, 5).

One of the most studied interactions is that of estrogen and progesterone with regulators of the HPA-axis. Contraceptives containing these hormones (in the form of progestins and ethinyl estradiol) are thought to alter mineralcorticoid receptor (MR) action in regulating stress responses, thereby affecting emotion information processing, and to suppress or increase circulating cortisol levels through different phases of the menstrual cycle (2, 3, 20). In researching this relationship, MR haplotype (defined by SNPs rs2070951 and rs5522) was found to moderate the effect of combined oral contraceptives on emotional recognition and memory (2). Carriers of MR haplotypes 1 or 3 were found to be more sensitive to the effects of HCs on negative emotional memory and perception-bias (2). Participants with these haplotypes taking HCs were more aware of sad or fearful faces, a finding previously recognized in patients with depression (2). However, follow-up studies showed that haplotype was not significant in moderating HC effect on affect itself (3).

Another HPA regulator, the FKBP5 protein (which inhibits the role of glucocorticoid receptors in stress response to the HPA cycle), is known to have upregulated expression in high-cortisol environments (20). Hertel et al. found that while circulating cortisol levels were higher in oral contraceptive users (a known effect of combined HCs), *FKBP5* transcription was only upregulated in a subset of individuals. They postulated that an *FKBP5* SNP (rs1360780) could modulate the effect of oral contraceptives and high cortisol on expression. Although they were not able to show a direct effect of this SNP on depressive outcomes, they proposed that specific FKBP5 genotypes may help identify patients at high risk for HC-related mood disorders (20).

Beyond the HPA axis, pharmacogenomic associations have been found between HCs, startle response, and circadian rhythm, potentially influencing perceived energy and stress. The *CLOCK* gene, which plays a central role in circadian rhythm and is implicated in numerous psychiatric disorders, was studied by Armbruster et al. and found to have an interaction effect with hormone status. SNP rs1801260 is linked with evening wakefulness and sleep dysregulation, as well as possible affective changes (21). In one subset of their study, Armbruster et al. specifically tested reaction to emotional stimuli and circadian rhythms in patients taking combined oral contraceptives compared with free-cycling participants separated by *CLOCK* genotype. It was found that while certain measures of startle response (increased skin conductance and decreased corrugator activity) was stronger in homozygous carriers of the T allele in naturally-cycling users, these trends reversed in HC users (21). HC users also exhibited less differential diurnal preferences based on genotype than non-users (21). The differential physiological stress response and reported sleep preferences may indicate that in some users, HCs counteract natural energy fluctuations and stress response mechanisms. New users adjusting to these changes might notice these side effects as altered mood, affect changes, or fatigue.

Overall, there is compelling, yet limited, evidence that genotype can compound the effect of hormone status on mood and energy levels. Certain combinations of alleles might make some women more likely to experience unpleasant mood alterations, depression, or sleep disturbances while taking HCs. Identifying the link between specific HCs and varied emotional lability is difficult in experimental research, however. One limitation is that contraceptives were generally not compared within these studies, with the majority of participants taking combined oral contraceptives.

## Hypertension

While HCs are generally associated with an increased risk of hypertension, the mechanism is not well understood, and a complex interplay between steroid hormones, the renin-angiotensin system (RAS), and blood pressure is inferred (11). The angiotensinogen (*AGT*) gene and *ESR* genes may all be involved in the etiology of HC-induced hypertension (11, 22). Angiotensinogen levels are found to be higher in women using HCs, as estrogens induce production in the liver, likely driving increased vasoconstrictor production (11, 22). Two polymorphisms on the angiotensinogen gene (rs699 and promoter region variants) and one on the angiotensin I converting enzyme gene (an insertion/deletion polymorphism) have been researched as possible links to the physiological pathways behind HC-induced hypertension (11). In women with HC-induced hypertension compared with women with essential hypertension, the frequency of the rs699T allele was higher, a difference which increased when stratified by the insertion/deletion polymorphism on the *ACE* gene (11). This suggests not only genetic predisposition to angiotensinogen dysregulation following exogenous estrogen introduction, but also that numerous SNPs interact to produce this predisposition, implying a complex, multifactorial genetic risk among the RAS alone.

Further complicating the system, *ESR1* and *ESR2* may also contribute to the pathology of this HC side effect. Estrogen receptors present on smooth muscle cells are known to regulate vasodilatory and vasoconstrictive proteins in these tissues (22). At least one *ESR2* polymorphism (rs1256049) has been shown to have an interaction effect with combined oral contraceptive use, as the heterozygote variant genotype (G/A) was found to be significantly associated with hypertension, a trend amplified in the population of subjects using HCs (22). Furthermore, cumulative combined oral contraceptive use of 15 years or more was found to have an increased effect on hypertension risk, meaning that not only did combined oral contraceptive use affect hypertension, but risk was dependent on duration of exposure. The recessive genotype (A/A) was only slightly associated with hypertension and was not a significant risk factor, nor was a second *ESR2* SNP (rs4986938) (22).

The multifactorial risk profile demonstrates the complexity inherent to pharmacogenomic interactions. While evidence suggests certain polymorphisms have a role in the development of hypertension, additional SNPs on related genes, the interaction effect between these genotypes, and drug-exposure length could all contribute to the complex picture of hormone-induced hypertension, making it extremely difficult to get a comprehensive picture of the syndrome. Blood pressure is easily monitored in most clinical settings, which could allow for early detection of HC-induced hypertension and medication discontinuation, although without additional research, it may be difficult to pinpoint which medications would be the safest alternatives, or which patients are at the highest risk prior to initiation.

## Additional side effects linked to HC usage

In addition to the side effects reviewed above, HCs have been implicated in many serious disease processes, including thrombus formation, stroke risk, and breast cancer development (4, 6). Some work has been done on gene/hormone-status interactions in relation to breast cancer and deep vein thrombosis, implicating variants on the *BRCA1* and the *F5* genes as risk factors (23, 24). While increased risk in any one of these conditions may be of serious medical consequence, these variants were not covered in depth by this review as our focus was common, burdensome side-effects. These more serious, life-threatening conditions may not produce symptoms until significant disease progression occurs and patients are generally counselled against certain HCs prior to initiating based on personal and family history (4, 6, 25). However, the potential for a joint effect of genetic and environmental (contraceptive use) risk factors in influencing these significant health risks bears further study and clinical consideration.

In contrast, additional common side effects, such as migraines, fatigue, and irregular vaginal bleeding are major sources of medication discontinuation (5, 26). Unfortunately, the scarcity of research into genetic risk factors for these symptomologies limited their contribution to this review. Recent work in transcriptomics does indicate differential expression of *CXCL9* and *TIMP1* plays a role in timing and severity of abnormal uterine bleeding in conjunction with HC use (26). Expanding the current body of work into genomics may help explain additional variance between side effect profiles. The contribution of these side effects to HC discontinuation should not be overlooked and holds potential as future avenues of study.

## Conclusion

This narrative review synthesizes the current evidence on genetic variation associated with hormonal contraceptive side effects and identifies key gaps for future research. Although there is a significant body of work supporting the role of pharmacogenetic contributions to side effects in HC use, this remains a broad and under-researched topic, limiting our ability to understand the intricacies of individual drug response. The diversity of contraceptive options and the large number of genes that may contribute to side effects are obstacles to efforts to build a more comprehensive systematic review. The strength of this narrative review is our ability to integrate heterogeneous early-stage findings from pharmacogenetics studies that are not yet suited to systematic review methods. The primary limitation is the restricted scope of available literature, which limits the strength of our conclusions.

Despite these challenges, it is important to predict side-effect risks to the best of our ability, as trial and error with contraceptive prescriptions carries risks of discontinuation, unwanted pregnancies, patient discomfort, and even severe health risks. This narrative review offers a glimpse at the current state of research and highlights the importance of further study in examining the complex relationship between hormonal birth control and genetics, supporting the notion that their interactions alter side effect profiles in patients. Future directions may include large-scale, genome-wide association studies and considerations of hormone status in other groups, such as post-menopausal women or pregnant women.
