# Supplementary material for: Genetic variation associated with side effects of hormonal contraception exposure: a narrative review
Source: Front Reprod Health. 2025 Nov 14;7:1720994. doi: 10.3389/frph.2025.1720994 (PMC12660243; doi:10.3389/frph.2025.1720994)
Supplement: Supplementary file 1 [file Table1.docx]

APPENDIX

Supplementary Table 1. General Baseline search

| Search Key | Key Concepts | Database | Results |
| --- | --- | --- | --- |
| ((estrogen) OR (progestin)) AND ('birth control' OR contraceptive) (side effects)  Filtered for full text availability and clinical trials | Hormonal Contraceptives and side effects | PubMed | 702 |

Supplementary Table 2. Abdominal/Pelvic Pain

| Search Key | Key Concepts | Database | Results |
| --- | --- | --- | --- |
| ('single nucleotide polymorphism'/exp OR 'genetic variation'/exp OR 'gene polymorphism'/exp OR 'genetic marker'/exp OR snp*:ti,ab OR polymorphism*:ti,ab OR 'genetic variant*':ti,ab OR 'genetic marker*':ti,ab OR 'genotype*':ti,ab) AND  ('hormonal contraceptive agent'/exp OR 'hormonal contraception'/exp OR 'oral contraceptive agent'/exp OR 'birth control pill':ti,ab OR 'oral contraceptive*':ti,ab OR 'hormonal contraceptive*':ti,ab OR 'combined oral contraceptive*':ti,ab OR 'progestin only pill*':ti,ab) AND  ('adverse drug reaction'/exp OR 'adverse effect*':ti,ab OR 'side effect*':ti,ab OR 'drug reaction*':ti,ab OR 'abdominal pain'/exp OR 'pelvic pain'/exp OR 'pelvic pain':ti,ab OR 'abdominal pain':ti,ab OR 'pelvic discomfort':ti,ab OR 'lower abdominal pain':ti,ab OR 'lower pelvic pain':ti,ab) AND  ('female'/exp OR women:ti,ab OR woman:ti,ab OR female:ti,ab) NOT ('male'/exp OR male:ti,ab) | Genetic factors  Hormonal Contraceptives  Abdominal or pelvic pain/adverse effects  In female patients | Embase | 74 |
| ('single nucleotide polymorphism'/exp OR 'genetic variation'/exp OR 'gene polymorphism'/exp OR 'genetic marker'/exp OR snp*:ti,ab OR polymorphism*:ti,ab OR 'genetic variant*':ti,ab OR 'genetic marker*':ti,ab OR 'genotype*':ti,ab) AND  ('hormonal contraceptive agent'/exp OR 'hormonal contraception'/exp OR 'oral contraceptive agent'/exp OR 'birth control pill':ti,ab OR 'oral contraceptive*':ti,ab OR 'hormonal contraceptive*':ti,ab OR 'combined oral contraceptive*':ti,ab OR 'progestin only pill*':ti,ab) AND  ('pain':ti,ab OR 'abdominal pain'/exp OR 'pelvic pain'/exp OR 'pelvic pain':ti,ab OR 'abdominal pain':ti,ab OR 'pelvic discomfort':ti,ab OR 'lower abdominal pain':ti,ab OR 'lower pelvic pain':ti,ab) AND  ('female'/exp OR women:ti,ab OR woman:ti,ab OR female:ti,ab) NOT ('male'/exp OR male:ti,ab) | Genetic factors  Hormonal Contraceptives  Abdominal or pelvic pain  In female patients | Embase | 41 |
| ( "Polymorphism, Single Nucleotide"[MeSH Terms] OR "Genetic Variation"[MeSH Terms] OR "Genetic Markers"[MeSH Terms] OR "Genotype"[MeSH Terms] OR polymorphism*[tiab] OR snp*[tiab] OR "genetic variant*"[tiab] OR "genetic marker*"[tiab] OR genotype*[tiab]  ) AND  ( "Contraceptives, Oral, Hormonal"[MeSH Terms] OR "Contraceptive Agents, Female"[MeSH Terms] OR "Birth Control Pills"[tiab] OR "oral contraceptive*"[tiab] OR "hormonal contraceptive*"[tiab] OR "combined oral contraceptive*"[tiab] OR "progestin only pill*"[tiab]  ) AND  ("Abdominal Pain"[MeSH Terms] OR "Pelvic Pain"[MeSH Terms] OR "abdominal pain"[tiab] OR "pelvic pain"[tiab] OR "lower abdominal pain"[tiab] OR "lower pelvic pain"[tiab] OR "pelvic discomfort"[tiab]  )AND  ( "Female"[MeSH Terms] OR women[tiab] OR woman[tiab] OR female[tiab]  ) NOT  ( "Male"[MeSH Terms] OR male[tiab]) | Genetic factors  Hormonal Contraceptives  Abdominal or pelvic pain  In female patients | PubMed | 14 |
| TS=( ("single nucleotide polymorphism" OR "genetic variation" OR "gene polymorphism" OR "genetic marker" OR snp* OR polymorphism* OR "genetic variant*" OR "genetic marker*" OR genotype*) AND  ("hormonal contraceptive agent" OR "hormonal contraception" OR "oral contraceptive agent" OR "birth control pill" OR "oral contraceptive*" OR "hormonal contraceptive*" OR "combined oral contraceptive*" OR "progestin only pill*" OR estrogen) AND  ("pain" OR "abdominal pain" OR "pelvic pain" OR "pelvic discomfort" OR "lower abdominal pain" OR "lower pelvic pain") AND  (female OR woman OR women) ) | Genetic factors  Hormonal Contraceptives  Abdominal or pelvic pain  In female patients | Web of Science | 20 |

Supplementary Table 3. Mood Changes

| Search Key | Key Parts | Database | Results |
| --- | --- | --- | --- |
| ('single nucleotide polymorphism'/exp OR 'genetic variation'/exp OR 'gene polymorphism'/exp OR 'genetic marker'/exp OR snp*:ti,ab OR polymorphism*:ti,ab OR 'genetic variant*':ti,ab OR 'genetic marker*':ti,ab OR 'genotype*':ti,ab) AND  ('hormonal contraceptive agent'/exp OR 'hormonal contraception'/exp OR 'oral contraceptive agent'/exp OR 'birth control pill':ti,ab OR 'oral contraceptive*':ti,ab OR 'hormonal contraceptive*':ti,ab OR 'combined oral contraceptive*':ti,ab OR 'progestin only pill*':ti,ab) AND  ('depression'/exp OR 'depressive disorder'/exp OR 'mood disorder'/exp OR depression:ti,ab OR depressive:ti,ab OR 'mood change*':ti,ab OR 'mood disorder*':ti,ab OR 'emotional side effect*':ti,ab OR 'mental health':ti,ab) AND  ('female'/exp OR women:ti,ab OR woman:ti,ab OR female:ti,ab) NOT ('male'/exp OR male:ti,ab) | Genetic factors  Hormonal Contraceptives  Mood effects  In female patients | Embase | 46 |
| ( "Polymorphism, Single Nucleotide"[MeSH Terms] OR "Genetic Variation"[MeSH Terms] OR "Genetic Markers"[MeSH Terms] OR "Genotype"[MeSH Terms] OR polymorphism*[tiab] OR snp[tiab] OR "genetic variant*"[tiab] OR "genetic marker*"[tiab] OR genotype*[tiab] ) AND  ( "Contraceptives, Oral, Hormonal"[MeSH Terms] OR "Contraceptive Agents, Female"[MeSH Terms] OR "Birth Control Pills"[tiab] OR "oral contraceptive*"[tiab] OR "hormonal contraceptive*"[tiab] OR "combined oral contraceptive*"[tiab] OR "progestin only pill*"[tiab] ) AND  ( "Depression"[MeSH Terms] OR "Depressive Disorder"[MeSH Terms] OR "Mood Disorders"[MeSH Terms] OR depression[tiab] OR depressive[tiab] OR "mood change*"[tiab] OR "mood disorder*"[tiab] OR "emotional side effect*"[tiab] OR "mental health"[tiab] ) AND  ( "Female"[MeSH Terms] OR women[tiab] OR woman[tiab] OR female[tiab] ) NOT ( "Male"[MeSH Terms] OR male[tiab] ) | Genetic factors  Hormonal Contraceptives  Mood effects  In female patients | PubMed | 12 |
| TS=( ("single nucleotide polymorphism" OR "genetic variation" OR "gene polymorphism" OR "genetic marker" OR snp* OR polymorphism* OR "genetic variant*" OR "genetic marker*" OR genotype*) AND  ("hormonal contraceptive agent" OR "hormonal contraception" OR "oral contraceptive agent" OR "birth control pill" OR "oral contraceptive*" OR "hormonal contraceptive*" OR "combined oral contraceptive*" OR "progestin only pill") AND  ('depression' OR 'depressive disorder' OR 'mood disorder' OR 'depression' OR 'depressive' OR 'mood change' OR 'mood disorder' OR 'emotional side effect*' OR 'mental health') AND  (female OR woman OR women) ) | Genetic factors  Hormonal Contraceptives  Mood effects  In female patients | Web of Science | 20 |

Supplementary Table 4. Fatigue

| Search Key | Key Parts | Database | Results |
| --- | --- | --- | --- |
| ('single nucleotide polymorphism'/exp OR 'genetic variation'/exp OR 'gene polymorphism'/exp OR 'genetic marker'/exp OR snp*:ti,ab OR polymorphism*:ti,ab OR 'genetic variant*':ti,ab OR 'genetic marker*':ti,ab OR 'genotype*':ti,ab) AND  ('hormonal contraceptive agent'/exp OR 'hormonal contraception'/exp OR 'oral contraceptive agent'/exp OR 'birth control pill':ti,ab OR 'oral contraceptive*':ti,ab OR 'hormonal contraceptive*':ti,ab OR 'combined oral contraceptive*':ti,ab OR 'progestin only pill*':ti,ab) AND  (fatigue:ti,ab OR tiredness:ti,ab OR exhaustion:ti,ab OR lethargy:ti,ab OR 'lack of energy':ti,ab OR 'low energy':ti,ab) AND  ('female'/exp OR women:ti,ab OR woman:ti,ab OR female:ti,ab) NOT ('male'/exp OR male:ti,ab) | Genetic factors  Hormonal Contraceptives  Fatigue  In female patients | Embase | 6 |
| ( "Polymorphism, Single Nucleotide"[MeSH Terms] OR "Genetic Variation"[MeSH Terms] OR "Genetic Markers"[MeSH Terms] OR "Genotype"[MeSH Terms] OR polymorphism*[tiab] OR snp[tiab] OR "genetic variant*"[tiab] OR "genetic marker*"[tiab] OR genotype*[tiab] ) AND  ( "Contraceptives, Oral, Hormonal"[MeSH Terms] OR "Contraceptive Agents, Female"[MeSH Terms] OR "Birth Control Pills"[tiab] OR "oral contraceptive*"[tiab] OR "hormonal contraceptive*"[tiab] OR "combined oral contraceptive*"[tiab] OR "progestin only pill*"[tiab] ) AND  ( fatigue OR tiredness OR exhaustion OR lethargy OR "lack of energy"[tiab] OR "low energy"[tiab]) AND  ( "Female"[MeSH Terms] OR women[tiab] OR woman[tiab] OR female[tiab] ) NOT ( "Male"[MeSH Terms] OR male[tiab] ) | Genetic factors  Hormonal Contraceptives  Fatigue  In female patients | PubMed | 1 |
| TS=( ("single nucleotide polymorphism" OR "genetic variation" OR "gene polymorphism" OR "genetic marker" OR snp* OR polymorphism* OR "genetic variant*" OR "genetic marker*" OR genotype*) AND  ("hormonal contraceptive agent" OR "hormonal contraception" OR "oral contraceptive agent" OR "birth control pill" OR "oral contraceptive*" OR "hormonal contraceptive*" OR "combined oral contraceptive*" OR "progestin only pill*") AND  ('fatigue' OR 'tiredness' OR 'exhaustion' OR 'lethargy' OR 'lack of energy' OR 'low energy') AND  (female OR woman OR women) ) | Genetic factors  Hormonal Contraceptives  Fatigue  In female patients | Web of Science | 0 |
| ('hormonal contraceptive agent'/exp OR 'hormonal contraception'/exp OR 'oral contraceptive agent'/exp OR 'birth control pill':ti,ab OR 'oral contraceptive*':ti,ab OR 'hormonal contraceptive*':ti,ab OR 'combined oral contraceptive*':ti,ab OR 'progestin only pill*':ti,ab) AND  (fatigue:ti,ab OR tiredness:ti,ab OR exhaustion:ti,ab OR lethargy:ti,ab OR 'lack of energy':ti,ab OR 'low energy':ti,ab) AND  ('female'/exp OR women:ti,ab OR woman:ti,ab OR female:ti,ab) NOT ('male'/exp OR male:ti,ab) NOT 'case report' | Hormonal Contraceptives  Fatigue  In female patients  **Genetic factors dropped due to low result counts for this category* | Embase | 411 |

Supplementary Table 5. Chest Pain

| Search Key | Key Parts | Database | Results |
| --- | --- | --- | --- |
| ('single nucleotide polymorphism'/exp OR 'genetic variation'/exp OR 'gene polymorphism'/exp OR 'genetic marker'/exp OR snp*:ti,ab OR polymorphism*:ti,ab OR 'genetic variant*':ti,ab OR 'genetic marker*':ti,ab OR 'genotype*':ti,ab) AND  ('hormonal contraceptive agent'/exp OR 'hormonal contraception'/exp OR 'oral contraceptive agent'/exp OR 'birth control pill':ti,ab OR 'oral contraceptive*':ti,ab OR 'hormonal contraceptive*':ti,ab OR 'combined oral contraceptive*':ti,ab OR 'progestin only pill*':ti,ab) AND  ('chest pain':ti,ab OR 'angina pectoris':ti,ab OR 'angina'/exp OR 'anginas' OR 'chest pressure'/exp OR 'thoracic pain'/exp) AND  ('female'/exp OR women:ti,ab OR woman:ti,ab OR female:ti,ab) NOT ('male'/exp OR male:ti,ab) | Genetic factors  Hormonal Contraceptives  Chest pain  In female patients | Embase | 4 |
| ("polymorphism, single nucleotide"[MeSH Terms] OR "Genetic Variation"[MeSH Terms] OR "Genetic Markers"[MeSH Terms] OR "Genotype"[MeSH Terms] OR "polymorphism*"[Title/Abstract] OR "snp"[Title/Abstract] OR "genetic variant*"[Title/Abstract] OR "genetic marker*"[Title/Abstract] OR "genotype*"[Title/Abstract]) AND  ("contraceptives, oral, hormonal"[MeSH Terms] OR "contraceptive agents, female"[MeSH Terms] OR "Birth Control Pills"[Title/Abstract] OR "oral contraceptive*"[Title/Abstract] OR "hormonal contraceptive*"[Title/Abstract] OR "combined oral contraceptive*"[Title/Abstract] OR "progestin only pill*"[Title/Abstract]) AND  ("chest pain"[MeSH Terms] OR "angina pectoris"[MeSH Terms] OR "angina"[All Fields] OR "angina pectoris"[All Fields] OR "anginas"[All Fields] OR "chest pressure"[All Fields] OR "thoracic pain"[All Fields]) AND  ("Female"[MeSH Terms] OR "women"[Title/Abstract] OR "woman"[Title/Abstract] OR "Female"[Title/Abstract]) NOT ("Male"[MeSH Terms] OR "Male"[Title/Abstract]) | Genetic factors  Hormonal Contraceptives  Chest pain  In female patients | PubMed | 0 |
| TS=( ("single nucleotide polymorphism" OR "genetic variation" OR "gene polymorphism" OR "genetic marker" OR snp* OR polymorphism* OR "genetic variant*" OR "genetic marker*" OR genotype*) AND  ("hormonal contraceptive agent" OR "hormonal contraception" OR "oral contraceptive agent" OR "birth control pill" OR "oral contraceptive*" OR "hormonal contraceptive*" OR "combined oral contraceptive*" OR "progestin only pill*") AND  ('chest pain' OR 'angina pectoris' OR 'angina' OR 'anginas' OR 'chest pressure' OR 'thoracic pain') AND  (female OR woman OR women) ) | Genetic factors  Hormonal Contraceptives  Chest pain  In female patients | Web of Science | 1 |
| ("contraceptives, oral, hormonal"[MeSH Terms] OR "contraceptive agents, female"[MeSH Terms] OR "Birth Control Pills"[Title/Abstract] OR "oral contraceptive*"[Title/Abstract] OR "hormonal contraceptive*"[Title/Abstract] OR "combined oral contraceptive*"[Title/Abstract] OR "progestin only pill*"[Title/Abstract]) AND  ("chest pain"[MeSH Terms] OR "angina pectoris"[MeSH Terms] OR "angina pectoris"[MeSH Terms] OR "angina"[All Fields] AND "pectoris"[All Fields] OR "angina pectoris"[All Fields] OR "angina"[All Fields] OR "anginas"[All Fields] OR "chest pressure"[All Fields] OR "thoracic pain"[All Fields]) AND  ("Female"[MeSH Terms] OR "women"[Title/Abstract] OR "woman"[Title/Abstract] OR "Female"[Title/Abstract]) NOT ("Male"[MeSH Terms] OR "Male"[Title/Abstract]) | Hormonal Agents  Chest Pain  In Women  **Genetic factors dropped due to low result counts for this category* | Pubmed | 52 |

Supplementary Table 6. Nausea and Vomiting

| Search Key | Key Parts | Database | Results |
| --- | --- | --- | --- |
| ('single nucleotide polymorphism'/exp OR 'genetic variation'/exp OR 'gene polymorphism'/exp OR 'genetic marker'/exp OR snp*:ti,ab OR polymorphism*:ti,ab OR 'genetic variant*':ti,ab OR 'genetic marker*':ti,ab OR 'genotype*':ti,ab) AND  ('hormonal contraceptive agent'/exp OR 'hormonal contraception'/exp OR 'oral contraceptive agent'/exp OR 'birth control pill':ti,ab OR 'oral contraceptive*':ti,ab OR 'hormonal contraceptive*':ti,ab OR 'combined oral contraceptive*':ti,ab OR 'progestin only pill*':ti,ab) AND  ('nausea':ti,ab OR 'vomiting':ti,ab OR 'emesis':ti,ab OR queasiness:ti,ab OR 'feeling sick':ti,ab OR 'morning sickness':ti,ab) AND  ('female'/exp OR women:ti,ab OR woman:ti,ab OR female:ti,ab) NOT ('male'/exp OR male:ti,ab) | Genetic factors  Hormonal Contraceptives  Nausea  In female patients | Embase | 5 |
| ("polymorphism, single nucleotide"[MeSH Terms] OR "Genetic Variation"[MeSH Terms] OR "Genetic Markers"[MeSH Terms] OR "Genotype"[MeSH Terms] OR "polymorphism*"[Title/Abstract] OR "snp"[Title/Abstract] OR "genetic variant*"[Title/Abstract] OR "genetic marker*"[Title/Abstract] OR "genotype*"[Title/Abstract]) AND  ("contraceptives, oral, hormonal"[MeSH Terms] OR "contraceptive agents, female"[MeSH Terms] OR "Birth Control Pills"[Title/Abstract] OR "oral contraceptive*"[Title/Abstract] OR "hormonal contraceptive*"[Title/Abstract] OR "combined oral contraceptive*"[Title/Abstract] OR "progestin only pill*"[Title/Abstract]) AND  ( "Nausea"[MeSH Terms] OR "Vomiting"[MeSH Terms] OR "Emesis"[MeSH Terms] OR nausea[tiab] OR vomiting[tiab] OR emesis[tiab] OR queasiness[tiab] OR "feeling sick"[tiab] OR "morning sickness"[tiab] ) AND  ("Female"[MeSH Terms] OR "women"[Title/Abstract] OR "woman"[Title/Abstract] OR "Female"[Title/Abstract]) NOT ("Male"[MeSH Terms] OR "Male"[Title/Abstract]) | Genetic factors  Hormonal Contraceptives  Nausea  In female patients | PubMed | 4 |
| TS=( ("single nucleotide polymorphism" OR "genetic variation" OR "gene polymorphism" OR "genetic marker" OR snp* OR polymorphism* OR "genetic variant*" OR "genetic marker*" OR genotype*) AND  ("hormonal contraceptive agent" OR "hormonal contraception" OR "oral contraceptive agent" OR "birth control pill" OR "oral contraceptive*" OR "hormonal contraceptive*" OR "combined oral contraceptive*" OR "progestin only pill*") AND  ('nausea' OR 'vomiting' OR 'emesis' OR 'queasiness' OR 'feeling sick' OR 'morning sickness') AND  (female OR woman OR women) ) | Genetic factors  Hormonal Contraceptives  Nausea  In female patients | Web of Science | 2 |
